# Evolving Sweet Preferences: Temporal Trends in Australian Non-Alcoholic Beverage Sales from 1997 to 2024

**DOI:** 10.3390/nu18020361

**Published:** 2026-01-22

**Authors:** Carlene S. Starck, Tim Cassettari, Emma Beckett, Flavia Fayet-Moore

**Affiliations:** 1 FOODiQ Global, Sydney, NSW 2000, Australia; tim@foodiq.global (T.C.); emma.beckett@acu.edu.au (E.B.); flavia@foodiq.global (F.F.-M.); 2 School of Behavioural and Health Sciences, Australian Catholic University, North Sydney Campus, Sydney, NSW 2060, Australia; 3 School of Environmental and Life Sciences, The University of Newcastle, Newcastle, NSW 2308, Australia

**Keywords:** water-based beverages, non-alcoholic beverages, sugar-sweetened beverages, non-sugar-sweetened beverages, unsweetened beverages, sugar contribution, soft drinks, volume sales, trends analysis

## Abstract

**Background/Objectives:** Understanding the purchasing behaviour of sweetened beverages is important, as beverages have been highlighted as a key target for reducing sugar intake. This research aimed to provide a comprehensive understanding of trends in per capita volume sales of non-alcoholic water-based beverages (WBB) in Australia and their contribution to dietary sugars between 1997 and 2024. **Methods:** Volume sales data for the years 2018 to 2024 (Circana Connect) were integrated with three previously published datasets spanning 1997 to 2018, with adjustments to reflect the total market where applicable. Per capita volume sales were determined using national population data (Australian Bureau of Statistics) for each corresponding year. Linear regression analysis was performed to assess trends in per capita volume sales over time. Sugar contributions of each beverage category were modelled based on representative sugar content data. **Results:** Total WBB sales showed consistent growth over the 28-year period (1.68 L/person/year, 36.2%). Within this, sales of sugar-sweetened beverages (SSB) declined (−1.08 L/person/year), with a concurrent increase in non-sugar-sweetened and unsweetened beverage purchases (2.74 L/person/year). This transition became more pronounced from 2015 and coincided with a decreased contribution of WBB to dietary sugars (−0.13 kg/person/year, *p* < 0.001). There was variation in sales and sugar contribution trends by beverage category. Functional beverages (e.g., coconut water, protein water) showed increases in sales and sugar contribution. **Conclusions:** The last 28 years have seen a trend in beverage purchases away from sugar-sweetened to non-sugar-sweetened and unsweetened varieties. This comprehensive analysis of consumer beverage choices makes a valuable contribution to policy and health-focused food industry initiatives.

## 1. Introduction

Excess dietary sugars intake, particularly that of added and free sugars, has been highlighted as contributing to the development of obesity and associated cardiometabolic diseases such as type 2 diabetes (T2D) and cardiovascular disease (CVD), as well as dental caries [1,2]. While global efforts to reduce added sugars intake have been made, with recommended targets for free sugar intake no higher than 5–10% of dietary energy, population-level intakes remain higher than recommended worldwide [3]. Within dietary sugar sources, sugars provided by liquid forms have been reported to have a greater adverse effect on health compared to those from solid forms, including a greater risk for weight gain, insulin resistance, and metabolic syndrome [4]. As SSBs remain the largest contributor to dietary free sugars in Australia [5], beverages have been highlighted as a key target to reduce sugar intake and address the relationship between dietary sugars and chronic disease [6]. Both dietary intake and sales data regarding beverages provide information on the contribution of beverages to sugar intake. While beverage sales are not a direct reflection of intake (e.g., due to missing sales channels and wastage), these data are a valuable indicator of patterns in purchasing behaviour.

Previous analyses of trends in beverage purchases in Australia have shown reductions in SSB sales over time, with a concomitant increase in sales of non-sugar sweetened beverages (NSSBs) and plain water between 1997 and 2018 [7,8,9]. An analysis of sugar content across Australian beverage categories between 2015 and 2019 identified a 17% reduction in the mean sugar content of beverages overall, with the largest reductions in soft drinks and milk-based drinks (almost 30%) [10]. Recent data from the 2023 NNPAS indicates a steady decrease in SSB consumption and a small increase in NSSBs since 1995 [5]. These patterns correlate with initiatives to reduce dietary sugar intake from beverages, including public health efforts (such as educational awareness campaigns), and government policy and regulatory measures (including the Health Star Rating system and school canteen policies). More recently, industry initiatives such as the Australian Beverages Council (ABCL) Sugar Reduction Pledge have been implemented. Signatories of the pledge committed to reducing sugar content by 20% across their non-alcoholic beverage portfolios between 2015 and 2025, with an additional stretch target of 25% introduced in 2022. Despite evidence of ongoing shifts in both beverage consumption and within the beverage industry, there is a lack of data post-2018 on beverage sales in Australia, as well as the distribution of sales between varieties with and without sugar. An updated understanding of trends in beverage sales and their contribution of sugar in the Australian population is needed to inform ongoing health-focused policy and food industry efforts.

In addition to carbonated soft drinks, a selection of newly emerging beverage categories is focused on providing healthier drink options or functional outcomes, in line with a market shift driven by consumers seeking healthier and more functional drink options [11]. However, while many of these “functional beverages” contain low or no sugar, select varieties retain a moderate sugar content (such as coconut waters), with indications that this beverage category is experiencing rapid growth globally [11,12]. In addition, previous analyses of energy and sports drinks sales show positive growth for both sugar- and non-sugar- sweetened varieties [9]. Thus, despite the overall reduction in SSB sales, these growing markets have the potential to make a notable contribution to sugar intake and impact health over time, warranting specific consideration.

The primary objective of this research was to quantify 28-year trends (1997–2024) in per capita volume sales of non-alcoholic water-based beverages (WBB) in Australia and to estimate the contribution of these sales trends to dietary sugars. This analysis focuses primarily on volume sales of sugar-sweetened, non-sugar-sweetened, and unsweetened beverage categories. Secondary objectives include obtaining insight into the sales patterns of newer beverage categories (such as functional beverages) and additional sugar-containing beverages (flavoured milks, juices, and kombucha), and determining the contribution of different beverage categories to dietary sugars in the Australian population.

## 2. Materials and Methods

### 2.1. Overview of Included Beverages

This research integrates previously published data (from 1997 to 2018) with a new dataset from 2018 to 2024. The 1997 to 2018 dataset described seven individual categories of WBB (carbonated soft drinks, mineral waters, plain water, energy drinks, sports drinks, ready-to-drink teas, and mixers) with sub-categorisation based on the presence vs. absence of sugars, including sugar-sweetened (SS), non-sugar-sweetened (NSS, where non-nutritive sweeteners such as aspartame and stevia have been used), and unsweetened (UNS) beverages. Individual sweetener types included in NSS beverages could not be identified. The most recent dataset (2018–2024) introduced a new category of WBB, described as functional beverages (FNC), creating a total of eight WBB categories included as primary WBB in the current dataset. Functional beverages represent an emerging category of beverages, dominated by coconut water, with growing contributions from protein water, alkaline waters, and others. Mineral waters (1997 to 2018) were renamed as flavoured waters to better reflect the current market terminology and included beverages. A full description of each beverage category included within the primary WBB dataset is provided in Supplementary Table S1.

In addition to primary WBBs, additional beverages were added to the analysis series in the most recent publication [9], including fruit juices, flavoured milk-based beverages (such as chocolate milk), and kombucha. These could not be considered as primary WBBs due to the absence of data reflecting the size of the total market and were thus considered separately. These additional beverage categories were maintained within the 2018 to 2024 dataset and thus the current analysis, given their potential contribution to beverage-based sugar intake, and are described in Supplementary Table S1. In line with previous analyses, frozen drinks and cordials were not included.

Within each beverage category, sub-categorisation was based on sugar content. For primary WBB, sweetened and/or flavoured WBBs were classed as either sugar-sweetened (either with sugars added or containing inherent sugar, such as coconut water) or non-sugar-sweetened, with the latter representing both ‘diet’ and ‘no-joule’ varieties. Plain water was classed as either still or sparkling. Within additional beverages, flavoured milks and juices were classed as either ‘no added sugar’ or ‘added sugar’ varieties, due to all these beverages containing inherent sugars. Kombucha was treated in the same manner as WBBs (sugar-sweetened vs. non-sugar-sweetened). Sub-categories for each beverage category are included in Supplementary Table S1.

For some beverage categories and sub-categories, data spanning the entire 28-year period were not available. For example, data for functional beverages were limited to the 2018 to 2024 period, data for flavoured milks and juices were from 2009 to 2024, and data for kombucha were limited to 2015 to 2024. Available data for each beverage category and sub-category are presented in full within Supplementary Table S1.

As per previous analyses, umbrella groupings of beverages were based on sugar content and beverage type. These included total WBB, total sugar-sweetened WBB, total non-sugar-sweetened WBB, and total unsweetened WBB (including still, sparkling, and alkaline water). Total non-sugar-sweetened and unsweetened beverages were combined to form a fourth umbrella grouping defined as total no sugar beverages. A full description of each beverage umbrella grouping and the included categories and/or sub-categories is provided in Supplementary Table S2.

### 2.2. Dataset Development

Four distinct datasets, three of which have previously published and described [7,8,9], were combined by harmonising data across overlapping years. The previously published dataset (from 1997 to 2018) encompassing yearly volume sales (in litres) data was supplied by ABCL. This dataset has been described and assessed over a series of three published manuscripts in 2007 (1997 to 2006), 2014 (1997 to 2011), and 2020 (1997 to 2018). While detailed methods have been provided previously [7,8,9], in brief, data for the 2007 and 2014 analyses were sourced from AC Nielsen Scan Track surveys and adjusted to reflect total volume sales across all channels. The 2020 analysis involved a new dataset sourced from Information Resources, Inc. (IRI), Chicago IL, USA. The overlapping years from 2009 to 2011 were mapped to determine the level of adjustment required to harmonise the two datasets (1997 to 2011 and 2009 to 2018). This adjustment produced a series of estimates for the years 1997 to 2018 that were expected to reflect total volume sales for WBB across all channels. The dataset for additional beverages introduced in 2009 was not adjusted due to there being no equivalent data for harmonisation.

A similar harmonisation and adjustment process was conducted to incorporate the 2018 to 2024 data with the previous data to create the full 1997 to 2024 dataset. Data from 2018 to 2024 were sourced from Circana Connect (Sydney, NSW, Australia). These data cover all major grocery stores (including Coles, Woolworths, and independents, excluding ALDI) and convenience stores (including service stations) within Australia. The primary dataset used here includes a projection for ALDI supermarkets. The Circana Connect 2018 data were mapped to that from the previous 1997–2018 dataset to determine the level of adjustment needed for harmonisation across the two datasets. This was carried out by dividing the 2018 total sales value in litres of the previous 1997–2018 dataset by the 2018 total sales value in litres of the Circana Connect 2018–2024 dataset to produce a difference factor for each included WBB category. The final adjustment factor to be used across all WBB was defined as 1.4, which was the average of the difference factors across the WBB categories. As per the previous reports, this adjustment factor accounts for the fact that the Circana Connect dataset does not contain information on the totality of volume sales across all channels, such as additional convenience, foodservice, vending, and dining purchases. The calculated adjustment factor is consistent with a 2020 analysis of the distribution in beverage sales across different channels [13]. The final dataset is therefore an estimate of total volume WBB sales in Australia in litres per year from 1997 to 2024. As functional beverage varieties were included in the previous dataset, but categorised within the mineral water category, the 1.4 adjustment factor was also applied to functional beverages.

Data for additional beverages, including flavoured milks, juices, and kombucha, were harmonised with the previous dataset in a similar manner. As the 2018–2024 dataset for juices was limited to chilled varieties, representing a smaller proportion of the total market compared to flavoured milks and kombucha, separate adjustment factors were calculated for juices (2.0) and flavoured milks and kombucha (1.2).

### 2.3. Analysis of Sales Data and Longitudinal Trends

Data for volume sales in litres per year were converted to sales in litres per capita per year (L/capita/year) using national population figures provided by the Australian Bureau of Statistics (ABS) [14]. As yearly population data are presented per quarter, the number of residents within the population per year was taken to be the average of the four quarters per calendar year. Market share was calculated for each category (e.g., carbonated soft drinks) and sub-category (e.g., sugar-sweetened carbonated soft drinks) as a percentage of total WBB sales in L/capita/year for each of four time points: 1997, 2015, 2019, and 2024. Market share was also calculated for sugar- and non-sugar sweetened beverages within each category as a proportion of that category. The four time points were used to reflect the beginning and end of both the overall (1997–2024) and most recent (2019–2024) time series, as well as acknowledge that 2015 was the reported start of the voluntary ‘Sugar Reduction Pledge’ initiated by ABCL. Per capita volume sales were also calculated for additional beverages, but these were omitted from market share calculations due to the data for these beverages not being reflective of the total market.

Longitudinal trends in volume sales per capita were assessed for all beverage categories via linear regression analysis, with the beta-coefficient reflecting the annual change in absolute volume sales. Annual growth rates were calculated as described by Equation (1).

Annual Growth Rate (%/yr) = [((Ve − Vb)/Vb)/n] × 100(1)

where Ve = volume sales at the end of the period, Vb = volume sales at the beginning of the period, and n = number of years in the period.

Trends and growth rates were calculated for five time periods: 1997–2024, 1997–2018, 2019–2024, 1997–2014, and 2015–2024, to capture the full 28-year range, as well as be able to compare the time periods of the most recent vs. the previous datasets, and those before and following the initiation of the Sugar Reduction Pledge. While linear regression was used to summarise average annual trends within each period, segmented regression analyses were applied separately to formally assess whether the rate of change differed before and after the pre-specified breakpoints (2015 and 2019). Where data were missing for any beverage-time period combination (for example, functional beverages from 2018 to 2024), time periods were adjusted to reflect the available data. Linear regression analysis and comparison between temporal trends (e.g., 1997–2018 vs. 2019–2024) were performed using R software (version 4.2.2). Growth rates (percentage change) were calculated using Microsoft Excel software (version 2510).

### 2.4. Trends in Category-Specific Distribution of Sugar vs. No-Sugar Beverage Sales over Time

To investigate the distribution in sales of sugar vs. no-sugar WBB across different beverage categories, volume sales of each sub-category (sugar-sweetened vs. non-sugar sweetened) were expressed as a proportion of total volume sales for that category at four time points: 1997, 2015, 2019, and 2024. As for previous analyses, these four time points were selected to reflect the beginning and end of both the overall and most recent time series, as well as capture the initiation of the Sugar Reduction Pledge. An identical analysis was performed for additional beverages, with flavoured milks and juices separated according to beverages with and without added sugar. For additional beverages, the years of analysis were 2009, 2015, 2019, and 2024, to reflect the available data.

### 2.5. Analysis of Sugars Contribution

Sugar’s contribution by beverages to the Australian population (kg per capita per year) was modelled by adjusting the per capita volume sales for each beverage sub-category by the mean sugar content (in kilograms per litre) of that beverage sub-category for each year, as described by Equation (2).

Sugar’s contribution per capita (kg/capita/yr) = Vy × S(2)

where Vy = volume sales in L/capita for the year and S = sugar content in kg/L.

Sugar content estimates were based on those used in previous analyses [7,8,9], derived from Brix measurements. For beverage categories not covered by the previous Brix estimates (such as functional beverages and flavoured milk with no added sugar), sugar content was estimated by collecting available data for all listed beverages within the 2024 sales dataset, based on current nutritional information panel data reported on grocery and product websites, as applicable, and calculating the average of these values. Nutrition data was collected in September 2025 and therefore serves as a proxy for sugar content over the time series only. Sugar provided by total water-based beverages and total additional beverages was calculated by summing the sugars provided by the individual beverage categories, as a proportion of sales.

The sales-based sugars content of each total beverage category and grouping per year of the series in g/L was calculated by summing the sugars provided for the included subcategories (for example, sugar-sweetened and non-sugar sweetened varieties) in kg/capita/year, then dividing by the total sales for that category in L/capita/year and multiplying by 1000, as described by Equation (3).

Category sugars contenta (g/L/yr) = [(∑nb = 1 Sab)/Vy] × 1000(3)

where a = the beverage category (e.g., carbonated soft drinks), b = the beverage sub-category (e.g., sugar-sweetened), S = sugar (in kg/capita/year) provided by subcategory b of category a, and Vy = volume sales in L/capita for the year.

Trends in sugar contribution (kg/capita/year) and content (g/L/year) were calculated via linear regression analysis for the full 28-year time period from 1997 to 2024 for the major beverage groupings and categories.

To gain an understanding of the potential impact of product reformulation on sugar contribution in 1997 compared to that in 2024, data on sugar content were collected for all listed beverages within the 2024 sales dataset provided by Circana Connect, based on the current available nutritional information panel data reported on grocery and/or product websites, as applicable. The average of these values was used as an estimate for the sugar content of each beverage category. As the nutrition data were collected in October 2025, these values were used as a proxy to estimate the 2024 sugar content.

### 2.6. Additional Statistical Considerations

Statistical analyses were conducted using R (version 4.2.2) and Microsoft Excel (version 2510). For each linear regression analysis, the strength of the trend was assessed against the coefficient of determination (R^2^) and significance (*p*-value) of the linear fit, with significance defined as *p* < 0.05. Model fit was assessed using the Multiple R^2^ value, as only one predictor (year) was included in each model, allowing direct comparability across beverage categories. As these analyses were intended to describe directional trends rather than support causal inference or make predictions, model assumptions were not formally tested. However, given that annual time-series data may deviate from classical regression assumptions (e.g., residual normality/homoskedasticity and potential autocorrelation), and that a large number of related trend tests were performed, *p*-values were interpreted cautiously, and emphasis was placed on slope magnitude/direction and consistent patterns across categories and periods. As a sensitivity analysis for potential autocorrelation, standard errors were additionally estimated for a pre-specified set of primary series representing major beverage groupings (total WBB, total sugar-sweetened WBB, total non-sugar-sweetened WBB, total unsweetened WBB, and total no sugar WBB) using Newey–West heteroskedasticity- and autocorrelation-consistent (HAC) estimators (lag = 1). This did not alter the interpretation of trend direction or magnitude for any beverage grouping. To test whether beverage trends differed between two periods (1997–2018 vs. 2019–2024, and 1997–2014 vs. 2015–2024), segmented linear regression models were fitted in R. Year was treated as a continuous predictor variable, and a binary period indicator (e.g., pre- vs. post-breakpoint) and its interaction with year were included to compare slopes, based on the coefficient of the interaction term. A significant difference in the rate of change between the two periods was defined as *p* < 0.05.

## 3. Results

### 3.1. Volume Sales and Market Share

An overview of volume sales (in L) per capita for each beverage category, sub-category, and grouping for the years 1997, 2015, 2019, and 2024, as well as the expression of these values as a percentage of the total market for WBB (sum of all WBB sales), is shown in Table 1. Across the time series, the largest contributor to WBB sales was carbonated soft drinks, although this contribution saw a reduction of 27.6 L/capita from 1997 (99.4 L/capita and 83% of total WBB sales) to 2024 (71.8 L/capita and 44% of total WBB sales). In contrast, plain water increased over the time series from 4.8% (5.8 L/capita) to 36% (52.8 L/capita) of total WBB sales from 1997 to 2024, respectively, to become the second largest contributor to WBB sales in 2024. Other beverage categories ranged from 0.1 to 6.7% of WBB sales. The reciprocal pattern seen for carbonated soft drinks and plain water was mimicked by total sugar-sweetened WBB and total no-sugar WBB (sum of unsweetened and non-sugar sweetened WBB sales). While total sugar-sweetened WBBs were the largest contributor to WBB sales in 1997 (83.9 L/capita and 70% sales), by 2024 this had decreased to 57.3 L/capita and 35% of sales. In contrast, no sugar WBB sales increased from 30.4 L/capita in 1997 (30% of sales) to 105.8 L/capita in 2024 (65% of sales). The increase in no-sugar WBB sales was primarily driven by unsweetened beverages (55% of no-sugar beverages in 2024), including plain water (still or sparkling) and unsweetened alkaline water.

For additional beverages, fruit juices maintained the highest per capita volume sales over time, although total sales decreased between 2009 (18.1 L/capita) and 2024 (15.4 L/capita), with both flavoured milks and kombucha increasing over this time.

### 3.2. Trends in Category Volume Sales

**Figure 1:**
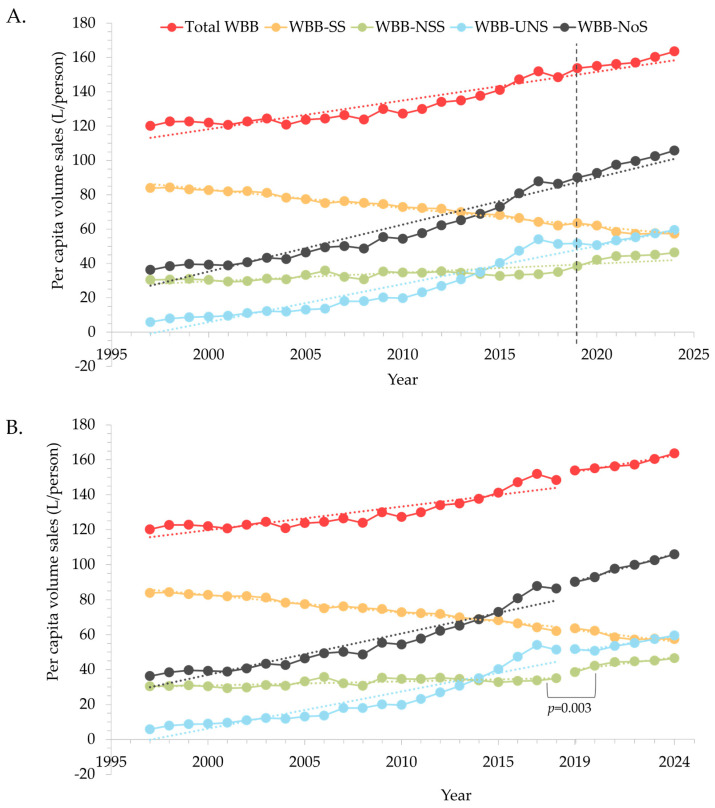
Change in yearly per capita volume sales (L/person) for major beverage groupings between 1997 and 2024, showing (**A**) non-segmented and (**B**) segmented analyses. Segmented models were used to compare the rate of change pre- vs. post-2019, with significance defined as *p* < 0.05. Total non-sugar sweetened beverages showed an increased rate of change from 2019 (*p* = 0.003). The dashed line in (**A**) represents the starting point of the most recent dataset (2019). NoS, no sugar (sum of non-sugar sweetened and unsweetened); NSS, non-sugar sweetened; SS, sugar-sweetened; WBB, water-based beverages; UNS, unsweetened.

Significant trends in per capita volume sales were observed across all beverage categories, with numerical data presented in Table 2. Overall sales of WBB grew at a rate of 1.68 L/person/year between 1997 and 2024, representing 36.2% growth over the 28-year period (Figure 1A). Alongside a steady decline in per capita sales of total sugar-sweetened WBB (−1.08 L/person/year), there was an almost 3-fold increase in the growth of total no sugar WBB (2.74 L/person/year). The largest contributor to the growth of no-sugar WBB was unsweetened WBB (primarily plain still/sparkling water, 2.22 L/person/year). Non-sugar-sweetened WBB grew 52% between 1997 and 2024. There was no change in growth rate for the most recent period (from 2019 to 2024) compared to the previous time period (1997 to 2018) for the majority of beverage groupings (Table 2, Figure 1B), with the exception of total non-sugar sweetened WBB, which experienced a significant increase post vs. pre 2019 (1.4 L/person/year vs. 0.24 L/person/year, *p* = 0.003, respectively). Large differences in sales growth occurred after 2015 (Table 2 and Table S3) for all beverage groupings except unsweetened beverages. The rate of growth for total WBB sales almost doubled (*p* < 0.001) post 2015, primarily due to an approximate 5-fold increase in sales for non-sugar-sweetened WBB. The rate of decline of sugar-sweetened WBB increased (*p* = 0.012).

**Figure 2:**
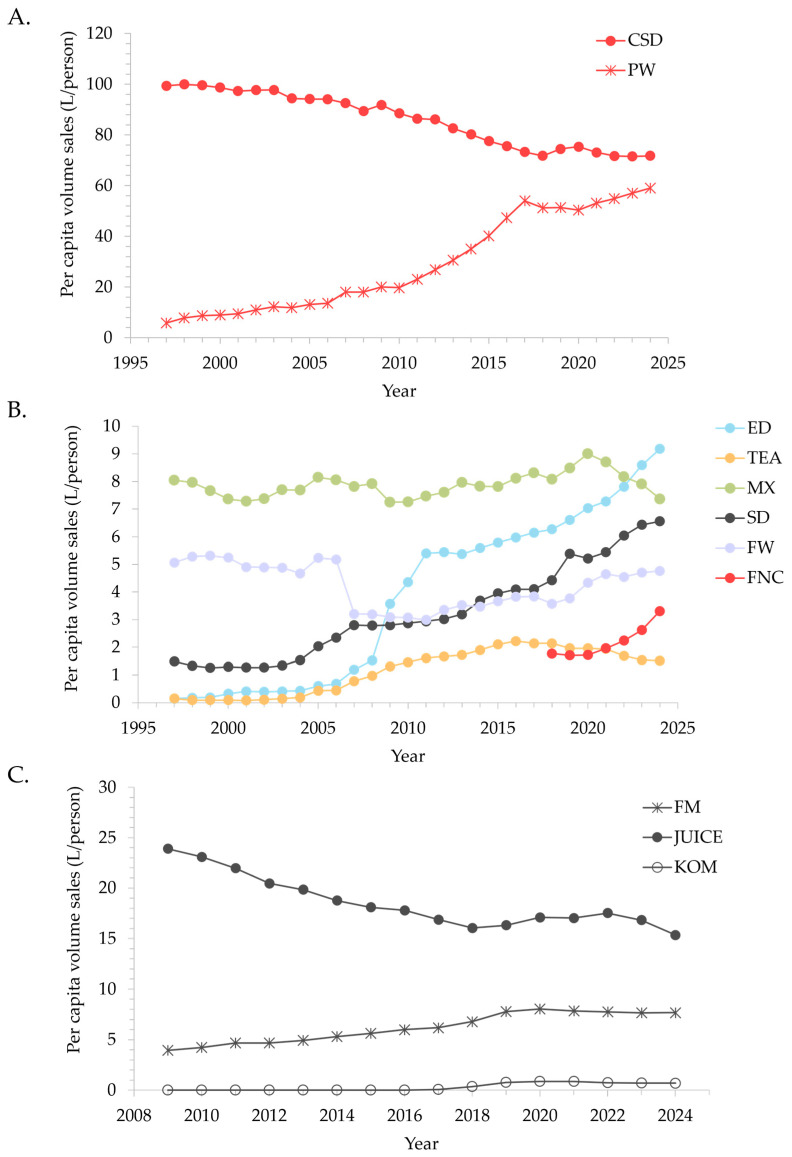
Change in yearly per capita volume sales (L/person) per category for (**A**) carbonated soft drinks and plain water from 1997 to 2024; (**B**) all other water-based beverages from 1997 to 2024; and (**C**) additional beverages from 2009 to 2024. CSD, carbonated soft drinks; ED, energy drinks; FM, flavoured milks; FW, flavoured waters; FNC, functional beverages; KOM, kombucha; MX, mixers; PW, plain water; SD, sports drinks; TEA, ready-to-drink teas.

Across individual beverage categories, there was notable variation over the 28-year period, with both positive (energy drinks, ready to drink teas, sports drinks, and plain water, all *p* < 0.001) and negative (carbonated soft drinks, mixers, and flavoured water, all *p* < 0.05) trends in sales (Table 2, Figure 2A,B). While the per capita volume sales of SSB sub-categories decreased for most beverage categories, the converse was observed for energy drinks, sports drinks, and functional beverages, with growth rates of 0.28, 0.17, and 0.21 L/person/year, respectively (all *p* < 0.005). Non-sugar sweetened beverages saw positive growth across all WBB categories, although the trend was not significant for flavoured water (0.006 L/person/year, *p* = 0.789). While data for functional beverage sales were limited to the 2018 to 2024 period, this category saw an 86% increase in growth (0.25 L/person/year), primarily due to sugar-sweetened and unsweetened varieties (rather than non-sugar sweetened varieties).

Similarly, trends for additional beverages were category dependent (Table 2, Figure 2C). Flavoured milk experienced steady growth in per capita sales from 2009 (0.3 L/person/year), driven primarily by flavoured milks without added sugar. While kombucha sales grew by 0.1 L/person/year from 2015 to 2024, this trajectory changed direction between 2019 and 2024 (−0.03 L/year, *p* = 0.012). In contrast, juice sales consistently declined from 2009 at a rate of 0.48 L/person/year.

For most beverage categories and sub-categories, the rate of growth in per capita volume sales (either positive or negative) was consistent between the previously published period (1997 to 2018) and that of the most recent dataset from 2019 to 2024 (Table 2 and Table S3). Exceptions included increased positive growth for non-sugar sweetened energy drinks and non-sugar sweetened sports drinks (both *p* < 0.001), while the rate of growth for sparkling water decreased (*p* < 0.001) but remained positive. For some beverages (total and sugar-sweetened ready-to-drink tea, and total mixers), the positive growth in sales observed between 1997 and 2018 was replaced by a steady decline in sales from 2019 onwards (all *p* ≤ 0.001). Flavoured milk and kombucha showed the same pattern (*p* < 0.05). Conversely, declining sales of flavoured water up to 2018 were replaced by positive growth from 2019 to 2024 (*p* = 0.048). Changes in growth post 2015 followed a similar pattern, with non-sugar sweetened carbonated soft drinks showing a significant increase in growth rate (*p* = 0.001) and non-sugar sweetened flavoured waters moving from decreasing to increasing sales (*p* < 0.001). In contrast, there was an increase in the rate of decline in sales of sugar-sweetened flavoured waters after 2015 (*p* < 0.001), and sugar-sweetened ready-to-drink (RTD) teas shifted from positive to negative growth pre- vs. post-2015 (*p* < 0.001). This was also seen for juices with added sugar (*p* = 0.002), while the decline in volume sales for juices with no added sugar slowed (*p* < 0.001). A heat map summarising the direction and magnitude of trends in volume sales per person per year for the total time period (1997–2024), as well as each segmented time period (1997–2014, 2015–2024, 1997–2018, 2019–2024), is provided as Supplementary Figure S1.

### 3.3. Distribution of Sugar vs. No-Sugar Beverage Sales

**Figure 3:**
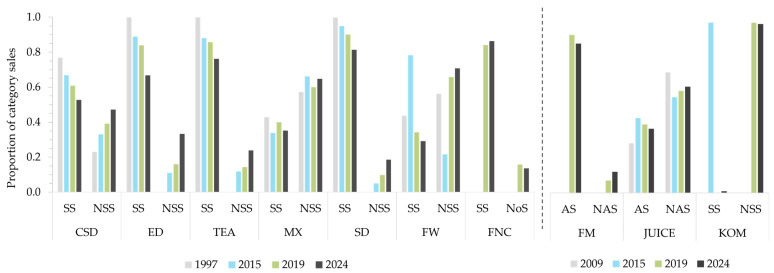
Distribution in sales by beverage subcategories with and without added sugar over time. Water-based beverages were assessed for the years 1997, 2015, 2019, and 2024. Additional beverages were assessed for the years 2009, 2015, 2019, and 2024. Limited data were available for functional beverages, flavoured milks, and kombucha, with results presented for those years with available data only. AS, added sugar; CSD, carbonated soft drinks; ED, energy drinks; FM, flavoured milks; FW, flavoured waters; FNC, functional beverages; KOM, kombucha; MX, mixers; NAS, no added sugar; NoS, no sugar (sum of non-sugar sweetened and unsweetened varieties); NSS, non-sugar sweetened; SD, sports drinks; SS, sugar-sweetened; TEA, ready to drink teas.

There was a consistent shift from sales of sugar-sweetened to non-sugar-sweetened beverage varieties (Figure 3). This pattern was also observed for beverages containing intrinsic sugars (flavoured milks and juices), despite an initial predominance of sales of juices with added sugars between 1997 and 2015. A similar trend was shown for flavoured waters, with an increased market share for sugar-sweetened varieties in 2015, followed by a trend away from sugar towards non-sugar-sweetened purchases in 2019 and 2024. For mixers, the shift in sales occurred in 2019. The only exception to these patterns was observed for functional beverages, with a small increase in sales for sugar-sweetened vs. no sugar (non-sugar-sweetened and unsweetened) varieties from 2019 to 2024.

### 3.4. Per Capita Contribution of Sugars from Beverages

**Figure 4:**
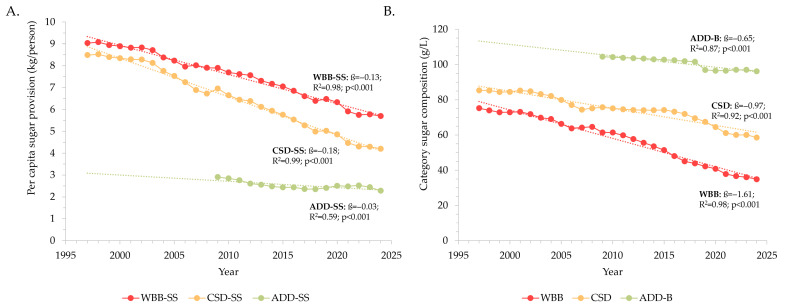
Sugar-related changes in major beverage groupings over time, shown as (**A**) per capita sugar contribution (kg/person), and (**B**) category sugar content (g/L). Each trend was assessed via linear regression, with *p* < 0.05 taken to reflect the presence of a significant linear trend. For each analysis, the slope of the trend (kg/person/year) is represented by the beta-coefficient (ß), the strength of fit by the coefficient of determination (R^2^), and significance was defined as *p* < 0.05. ADD-B, total additional beverages; ADD-SS, sugar-containing additional beverages (including those with intrinsic sugars); CSD, total carbonated soft drinks; CSD-SS, sugar-sweetened carbonated soft drinks; WBB, total water-based beverages; WBB-SS, sugar-sweetened water-based beverages.

**Figure 5:**
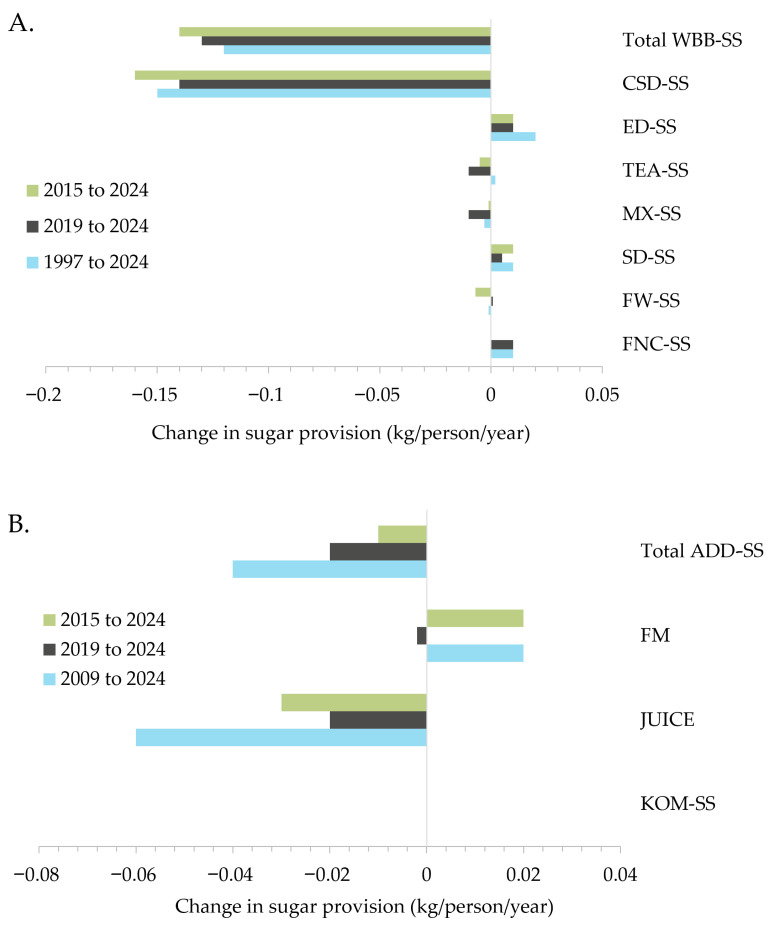
Change in per capita sugar contribution over time (kg/person/year) for applicable (**A**) water-based beverages, and (**B**) additional beverages. Changes for kombucha (KOM-SS) were negligible. ADD-SS, sugar-containing additional beverages (including those with intrinsic sugars); CSD, carbonated soft drinks; ED, energy drinks; FM, flavoured milks; FW, flavoured waters; FNC, functional beverages; KOM, kombucha; MX, mixers; SD, sports drinks; SS, sugar-sweetened; TEA, ready-to-drink teas; WBB-SS, sugar-sweetened water-based beverages.

Figure 4A shows a consistent and significant decrease in the per capita contribution of sugars from WBB sales from 1997 to 2024 (−0.13 kg/person/year, *p* < 0.001). This trend was driven by the sales of carbonated soft drinks, as the largest contributor to sugar-containing WBB (−0.18 kg/person/year, *p* < 0.001). Sugars provided by additional beverages also declined over time (−0.03 kg/person/year, *p* < 0.001). Similarly, the sugar content of total WBB and carbonated soft drinks decreased by 54% and 31% over time, respectively (Figure 4B). Additional beverages experienced a reduction in sugar content of −0.7 g/L/year (*p* < 0.001), equivalent to an 8% reduction between 2009 and 2024. Despite these overall decreases, some individual sugar-sweetened WBB and additional beverage categories showed consistent increases in the per capita contribution of sugars over time, including energy drinks, sports drinks, and functional beverages (Figure 5). While RTD teas (Figure 5A) and flavoured milks (Figure 5B) showed an overall increase in sugar contribution per year from 1997 to 2024 and 2009 to 2024, respectively, recent trends (from 2015 and 2019, respectively) indicate a decline in the sugars provided by these categories. In contrast, although relatively small, the contribution of sugar from flavoured waters showed a recent (from 2019) change from decreased to increased sugar contribution.

When current nutritional information panel data for sugar content were used to adjust per capita sugar contribution calculations for 2024, there was a change in both sugar content and per capita sugar contribution over time for most beverage categories (Table 3). Compared to unadjusted sugars content values used for the initial analyses, the sugars content per 100 mL was decreased for carbonated soft drinks (11.1 vs. 9.6 g/100 mL), RTD teas (6.7 vs. 4.6 g/100 mL), mixers (9.7 vs. 7.1 g/100 mL), flavoured milks (8.9 vs. 8.7 g/100 mL for varieties with added sugar and 4.1 g/100 mL with no added sugar), and juices (10.7 vs. 9.7 g/100 mL for varieties with added sugar and 8.9 g/100 mL with no added sugar). In contrast, the sugar content of energy drinks increased (10.9 vs. 11.5 g/100 mL), alongside that of flavoured waters (4.6 vs. 6.5 g/100 mL) and kombucha (2.5 vs. 3.0 g/100 mL). Sports drinks remained unchanged. Together, these differences resulted in an 11% decrease in the content of total sugar-sweetened WBB. Similarly, per capita sugar contribution between 1997 and 2024 was further reduced for total WBB compared to unadjusted analyses (Table 3, by 7%, equivalent to 20 g/person/year), with similar results for carbonated soft drinks, mixers, total additional beverages, and juice. While RTD teas and flavoured milks showed increases in sugar contribution over time, these percentage changes were reduced in magnitude following adjustment, by 240% and 17%, respectively. Energy drinks and kombucha showed the opposite effect, with increases of 240% and 320%, respectively.

## 4. Discussion

To our knowledge, this paper presents the longest global beverage trends analysis conducted to date for per capita WBB volume sales. The included data represent the integration of four datasets over a 28-year period from 1997 to 2024 for the Australian population. Results show that while overall per capita WBB volume sales increased over this period, the contribution of WBB to dietary sugars decreased. The data indicate that these trends are due primarily to a trend away from sales of sugar-sweetened or added sugar varieties to those that contain non-sugar sweetener (e.g., ‘diet’ soft drinks), are unsweetened/plain (e.g., plain water), or contain intrinsic sugars only (e.g., juice with no added sugar). While these trends have been ongoing since 1997, they have become more apparent since 2015, coinciding temporally with the Sugar Reduction Pledge of ABCL, aiming to reduce the level of sugars provided by beverages. For some beverage categories, the final six years (since 2019) saw even greater changes. Conversely, while playing a relatively minor role in overall beverage sales, select beverage categories saw an increase in per capita sales of sugar-sweetened varieties. These included both traditional beverages, such as energy drinks and sports drinks, as well as the emerging functional beverage category, suggesting that these categories may be less sensitive to current market trends towards sugar reduction. The analyses presented showcase an evolution in the Australian beverage industry over time, including changes in the purchasing patterns of consumers, essential data that can inform ongoing health-focused public policy and food industry efforts.

The observed major trends in per capita WBB volume sales in Australia (an overall increase in total sales as well as of unsweetened and non-sugar sweetened varieties, yet decreased sales of sugar-sweetened varieties) are consistent with those seen globally. In the UK, analysis of per capita volume sales of total soft drinks showed a 5% increase between 2015 and 2019, yet an overall 30% reduction in the sales of sugar from these products [15]. Analysis of per capita volume sales of sugary drinks in Canada found a 17 to 23% decrease between 2004 and 2015, depending on the sales dataset used [16]. In New Zealand, analysis of volume sales per household showed no change in total and unsweetened beverage purchases between 2015 and 2019, yet a decrease in sugar-sweetened beverage sales and an increase in artificially sweetened beverage and plain water sales [17]. These patterns also align with public health efforts to decrease the contribution of dietary free sugars from beverages. For example, the UK’s Soft Drink Industry Levy (SIDL) between 2015 and 2018 was suggested to be responsible for the observed reduction in sugar-sweetened soft drinks sales [15]. In the current analysis, comparison of trends before and after implementation of the Sugar Reduction Pledge in 2015 showed that the rate of decline in sugar-sweetened WBB sales increased by 50% from 2015 to 2024, while both per capita non-sugar sweetened WBB and no sugar (non-sugar sweetened plus unsweetened). WBB volume sales increased by at least 100%. For non-sugar-sweetened WBB, the rate from 2019 to 2024 was over fivefold greater than that before 2019, suggesting a particular increase in growth of these beverages in the most recent period. Overall, these findings indicate that efforts to reduce the consumption of sugar-sweetened beverages across multiple initiatives and promote non-sugar alternatives have been successful.

While beverages remain the primary contributor to free sugar intake in Australia [5], the current findings suggest that the contribution of sugars from water-based and additional (e.g., juice, flavoured milk) beverages has been decreasing in a meaningful manner since 1997 and 2009, respectively, with a slight increase in the rate of decline for WBB in the last ten years. Similarly, based on the available data, the estimated sugar content of total WBB and carbonated soft drinks available in the Australian market has decreased by around 55% and 30%, respectively. Even the sugar content of total additional beverages, many of which contain intrinsic sugars, appears to have decreased by almost 20%. In line with this, a recent retrospective analysis of the sugar content of non-alcoholic beverages in Australia between 2015 and 2019, conducted using the FoodSwitch database, identified an overall 9% reduction in the mean sugar content of sugar-sweetened beverages, with the largest decreases in soft drinks (25%) and milk-based protein drinks (18%) [10]. These changes were attributed to both reformulation and increased sales of low or no-sugar beverages, as per the objectives of the Australian Sugar Reduction Pledge. These results further correlate with data showing that the mean free sugar intake in the Australian population has reduced over time to currently sit below 10% of energy, according to the most recent nutrition survey data [18], and are consistent with WHO targets. Alongside a decrease in mean total free sugar intake since 1995 (66 g down to 43.4 g in 2023), the level of free sugar intake from beverages has also decreased (29 g down to 12.5 g) [5,19,20]. While sales data do not necessarily reflect consumption, the substantial role of beverages in free sugar intake suggests that the observed reductions in beverage sugar content and contribution may have contributed to these reductions. To contextualise these findings, it is useful to consider a counterfactual scenario in which sugar content and the distribution of beverage purchases remained at baseline levels, while total per capita beverage volume sales followed the observed trends. Under such a scenario, sugar’s contribution from beverages would have increased in parallel with rising total beverage purchases. The divergence between the observed and counterfactual scenarios is consistent with the combination of declining sugar content within beverage categories and shifts in purchasing away from sugar-sweetened beverages toward non-sugar-sweetened and unsweetened varieties. Beyond alignment with WHO free sugars targets, the observed shift from sugar-sweetened toward non-sugar-sweetened and unsweetened beverages is plausibly relevant to both metabolic and oral health. Systematic reviews with meta-analysis indicate that replacing sugar-sweetened beverages with non-sugar-sweetened or unsweetened alternatives is associated with reductions in bodyweight/BMI and modest improvements in cardiometabolic risk factors [21,22,23]. For oral health, sugar-sweetened beverage consumption is consistently associated with increased dental caries risk, whereas this relationship is generally not observed for non-sugar-sweetened/unsweetened varieties [24,25,26]. However, some non-sugar-sweetened carbonated beverages remain acidic and may contribute to dental erosion independent of sugar content, with erosion risk influenced by beverage chemistry, dietary behaviours, and host factors [27,28]. The comprehensive analysis presented here, in combination with Australian consumption patterns, highlights a trajectory of beverage consumption in line with the reduction in free sugar intakes. It is important to consider that some additional beverage categories provide essential nutrients to the diet, alongside intrinsic sugars, including juices (e.g., vitamin C, potassium, phytonutrients), and flavoured milks (e.g., calcium, vitamin A, potassium). While flavoured milks with no added sugar showed positive growth over the time series, sales of juices with no added sugar declined from 2009 to 2024. As these beverages have been reported to support improved nutritional status and diet quality in children and adults [29,30,31,32], additional understanding regarding how sales trends for these beverages translate to patterns of intake in key population groups is needed.

It is worth noting that some beverage categories did not follow the observed major trends. In particular, for energy drinks, sports drinks, and the emerging functional beverages category, both total beverage sales and those of sugar-sweetened varieties saw an increase in sales over time. Similar findings have been reported in Canada from 2004 to 2015 [16]. The observed divergences may imply that key beverages are at risk of increasing the contribution of the beverages category to sugar intake over time and thus require ongoing monitoring. However, these beverages contribute a small proportion of the total WBB market and have a small value compared to most other WBB (0.14 L/person) at the start of the time series (in 1997). In addition, the notable shift in the distribution of sales away from sugar-sweetened varieties towards non-sugar-sweetened or no-added sugar varieties, which has been the greatest and most consistent since 2015, indicates that there is a strong consumer selection towards no-sugar beverages regardless of beverage category. The only exception to these patterns is the emerging category of functional beverages, which showed growth of at least 80% between 2018 and 2024, regardless of sugar status. Due to the limited dataset available for this category, as well as the rapid growth across both sugar and non-sugar varieties, ongoing monitoring and measurement of sales and consumption behaviour may be warranted.

It is important to consider these findings within the context of the existing limitations of this research and the data. Further limitations to those already discussed include the categorisation of sold beverages (e.g., as carbonated soft drinks or energy drinks), which was undertaken by an independent body based on manual classification, introducing the potential for misclassification and associated error in estimates of total volume sales for each category and sub-category. Adjustment of the dataset to capture the total market was based on comparison to previous adjustments, and multiple datasets were harmonised to ensure consistency. For these reasons, per capita volume sales and all derived calculations should be interpreted as reliable estimates rather than exact measures. Per capita volume sales and sugar contribution estimates for additional beverages cannot be directly compared to those for WBB, as no total market sales estimate was available for adjustment. Data for the sugar content of each beverage category was derived from refractometric measurements (°Brix) of representative product samples collected almost two decades ago. This enabled comparison of recently collected data with earlier analyses, and the assessment of trends and changes in sugar contribution attributable solely to sales trends. However, as the sugar content of many beverage categories has declined over time, the values reported here may underestimate true reductions in the provision of beverages to dietary sugars. While a current sugar content dataset was created and highlighted such differences, these data are not directly comparable with Brix values, and additional research focused on the analysis of time-dependent trends in beverage sugar content is needed to understand the impact on beverage sugar contribution. Finally, the presented data and analyses do not account for differences in sales patterns between demographic groups. As such factors are likely to vary according to characteristics such as age, sex, and socioeconomic status, their inclusion in future assessments would be useful. Despite these limitations, the harmonisation procedures performed allowed for the integration of four distinct datasets to generate a robust 28-year time series that is the longest such analysis published to date. The included data are comprehensive and represent both grocery and convenience sales, as well as a wide range of beverage types with sub-categorisation based on sugar content. Together, these features provide a nuanced understanding of consumer beverage choices within the wider water-based beverages (WBB) category.

## 5. Conclusions

The Australian WBB industry has been experiencing steady growth over the 28-year period from 1997 to 2024. During this time, there has been a consistent movement away from purchases of sugar-sweetened to those of non-sugar-sweetened and unsweetened varieties, a pattern that appears to have accelerated in 2015, in alignment with the initiation of the industry’s Sugar Reduction Pledge. These sales trends correlate with reductions in sugar content and contribution, as well as patterns of beverage intake reported by national surveys, and thus may have played a role in the reduction in free sugar intake within the Australian population to below 10% of dietary energy. Some beverage categories, including energy drinks, sports drinks, and functional beverages, show increases in sugar-sweetened beverage sales over time, warranting ongoing monitoring. A key limitation in the presented analysis is the lack of accurate, sales-weighted sugar content data for each beverage category over time. While research to collate and analyse trends in the sugar content and true sugar contribution of beverage categories is needed, this research provides a robust estimate and the most comprehensive insight into consumer beverage purchases published to date. The current research can be used to inform public health, as well as policy and health-focused food industry initiatives.

## Figures and Tables

**Table 1:** Per capita sales ^1^ and market share of total and category-specific water-based and additional beverage categories.

Beverage Category	Per Capita Volume Sales (L/Person)				Market Share (%)			
	1997	2015	2019	2024	1997	2015	2019	2024
Water-based beverages								
Total water-based beverages (WBB)	120.10	141.11	153.76	163.52	100	100	100	100
Total Sugar-Sweetened (SS)	83.88	68.15	63.48	57.33	70	48	41	35
Total Non-Sugar Sweetened (NSS)	30.41	32.74	38.38	46.34	25	23	25	29
Total Unsweetened (UNS)	5.81	40.21	51.65	59.45	5	29	34	36
Total No Sugar (NoS = NSS + UNS)	36.21	72.96	90.03	105.79	30	52	59	65
Total carbonated soft drinks (CSD)	99.41	77.58	74.55	71.79	83	55	48	44
SS	76.45	51.88	45.29	37.87	64	37	29	23
NSS	22.96	25.7	29.16	33.92	19	18	19	21
Total energy drinks (ED)	0.14	5.79	6.61	9.18	0.1	4.1	4.3	5.6
SS	0.14	5.14	5.55	6.13	0.1	3.7	3.6	3.8
NSS	NA	0.64	1.06	3.06	NA	0.5	0.7	1.9
Total ready-to-drink teas (TEA)	0.15	2.1	1.96	1.51	0.1	1.5	1.3	0.9
SS	0.15	1.85	1.68	1.15	0.1	1.3	1.1	0.7
NSS	NA	0.25	0.28	0.36	0	0.2	0.2	0.2
Total mixers (MX)	8.04	7.81	8.49	7.37	6.7	5.5	5.5	4.5
SS	3.44	2.65	3.39	2.6	2.9	1.9	2.2	1.6
NSS	4.59	5.17	5.09	4.78	3.8	3.7	3.3	2.9
Total sports drinks (SD)	1.49	3.95	5.38	6.56	1.2	2.8	3.5	4.0
SS	1.49	3.76	4.84	5.34	1.2	2.7	3.2	3.3
NSS	0.003	0.2	0.53	1.22	0	0.1	0.3	0.8
Total flavoured waters (FW)	5.06	3.66	3.77	4.76	4.2	2.6	2.5	2.9
SS	2.21	2.86	1.29	1.39	1.8	2.0	0.8	0.9
NSS	2.85	0.79	2.48	3.37	2.4	0.6	1.6	2.1
Total plain waters (PW)	5.81	40.21	51.4	59.04	4.8	29	33	36
Still	5.81	35.51	45.65	52.75	4.8	25	30	32
Sparkling	NA	4.71	5.76	6.29	NA	3.3	3.7	3.9
Total functional beverages (FNC)	NA	NA	1.71	3.31	NA	NA	1.1	2.0
SS	NA	NA	1.43	2.85	NA	NA	0.9	1.8
NSS	NA	NA	0.02	0.04	NA	NA	0.01	0.03
UNS	NA	NA	0.25	0.41	NA	NA	0.16	0.25
Additional beverages								
Total flavoured milks (FM)	NA	5.61	7.79	7.67	NA	NA	NA	NA
Added sugars (AS)	NA	NA	7.21	6.72	NA	NA	NA	NA
No added sugars (NAS)	NA	NA	0.57	0.95	NA	NA	NA	NA
Total juices (JUICE)	NA	18.09	16.32	15.35	NA	NA	NA	NA
AS	NA	7.94	6.55	5.78	NA	NA	NA	NA
NAS	NA	10.15	9.77	9.57	NA	NA	NA	NA
Total kombucha (KOM)	NA	0.0004	0.77	0.69	NA	NA	NA	NA
SS	NA	0.0004	0.0009	0.006	NA	NA	NA	NA
NSS	NA	NA	0.77	0.69	NA	NA	NA	NA

^1^ Data from 1997 to 2024 were available for all WBB except functional beverages, for which data cover the period 2018 to 2024. Data for additional beverages covers the period 2009 to 2024 for flavoured milks and juice, while kombucha is limited to 2015 to 2024. NA, not applicable.

**Table 2:** Trends in volume sales per capita ^1^ for each beverage grouping, category, and sub-category from 1997 to 2024, and 2019 to 2024, and comparison of growth rates between time periods pre- and post-2019 and 2015.

Beverage	1997 to 2024					2019 to 2024					Comparison Between Periods ^2^ (*p*-Value)	
	Growth Rate			Trend		Growth Rate			Trend			
	%/p/per	%/p/y	L/p/y	R^2^	*p*	%/p/per	%/p/y	L/p/y	R^2^	*p*	Pre vs. Post 2019	Pre vs. Post 2015
Water-based beverages												
Total WBB	36.16	1.29	1.68	0.9	**<0.001**	6.35	1.06	1.88	0.93	**0.002**	0.586	**<0.001**
Total SS	−31.66	−1.13	−1.08	0.98	**<0.001**	−9.69	−1.62	−1.30	0.79	**0.019**	0.312	**0.012**
Total NSS	52.41	1.87	0.52	0.72	**<0.001**	20.75	3.46	1.40	0.86	**0.008**	**0.003**	**<0.001**
Total UNS	923.16	32.97	2.22	0.94	**<0.001**	15.09	2.51	1.75	0.92	**0.002**	0.754	0.850
Total NoS	193.25	6.9	2.74	0.95	**<0.001**	17.63	2.94	3.15	0.99	**<0.001**	0.479	**<0.001**
Total CSD	−27.78	−0.99	−1.26	0.95	**<0.001**	−3.57	−0.59	−0.74	0.76	**0.024**	0.267	**0.005**
SS	−50.46	−1.8	−1.59	0.99	**<0.001**	−16.37	−2.73	−1.54	0.89	**0.005**	0.941	0.542
NSS	47.72	1.7	0.33	0.64	**<0.001**	16.31	2.72	0.79	0.79	**0.019**	0.212	**0.001**
Total ED	6302.27	225.08	0.36	0.93	**<0.001**	38.88	6.48	0.52	0.97	**<0.001**	0.471	0.965
SS	4171.87	149	0.28	0.89	**<0.001**	10.36	1.73	0.11	0.86	**0.008**	0.264	**0.022**
NSS ^3^	29,892.69	1358.76	0.08	0.75	**<0.001**	188.22	31.37	0.41	0.97	**<0.001**	**<0.001**	**<0.001**
Total TEA	887.82	31.71	0.09	0.78	**<0.001**	−23.22	−3.87	−0.11	0.89	**0.005**	**<0.001**	**<0.001**
SS	650.8	23.24	0.07	0.74	**<0.001**	−31.85	−5.31	−0.12	0.93	**0.002**	**<0.001**	**<0.001**
NSS ^4^	930,959.04	37,238.36	0.02	0.91	**<0.001**	28.15	4.69	0.01	0.26	0.303	0.276	**0.012**
Total MX	−8.25	−0.29	0.02	0.19	**0.02**	−13.1	−2.18	−0.27	0.74	**0.029**	**0.001**	0.668
SS	−24.54	−0.88	−0.04	0.07	0.167	−23.43	−3.9	−0.17	0.94	**0.002**	0.640	0.835
NSS	3.96	0.14	0.06	0.18	**0.024**	−6.21	−1.04	−0.09	0.34	0.226	0.581	0.709
Total SD	339.21	12.11	0.2	0.94	**<0.001**	21.95	3.66	0.29	0.88	**0.006**	0.060	**<0.001**
SS	257.98	9.21	0.17	0.96	**<0.001**	10.14	1.69	0.13	0.79	**0.018**	0.796	0.084
NSS	42,377.69	1513.49	0.03	0.64	**<0.001**	129.18	21.53	0.16	0.90	**0.004**	**<0.001**	**<0.001**
Total FW	−5.79	−0.21	−0.04	0.15	**0.043**	26.49	4.42	0.17	0.74	**0.028**	**0.048**	**<0.001**
SS	−36.84	−1.32	−0.04	0.41	**<0.001**	7.97	1.33	0.01	0.02	0.790	0.898	**<0.001**
NSS	18.27	0.65	0.006	0.003	0.789	36.15	6.02	0.16	0.55	0.090	**0.033**	**<0.001**
Total PW	916.78	32.74	2.21	0.94	**<0.001**	14.85	2.48	1.71	0.92	**0.002**	0.732	0.972
Still	808.49	28.87	1.92	0.93	**<0.001**	15.56	2.59	1.60	0.93	**0.002**	0.852	0.506
Sparkling ^5^	206.33	11.46	0.27	0.9	**<0.001**	9.23	1.54	0.11	0.66	0.051	**<0.001**	**0.001**
Total FNC ^6^	86.35	12.34	0.25	0.82	**0.005**	93.7	15.62	0.31	0.90	**0.004**	NA	NA
SS	80.07	11.44	0.21	0.77	**0.010**	99.07	16.51	0.27	0.89	**0.004**	NA	NA
NSS	96.47	13.78	0.005	0.54	0.058	94.38	15.73	0.006	0.58	0.080	NA	NA
UNS	144.93	20.7	0.04	0.89	**0.001**	62.79	10.47	0.04	0.83	**0.012**	NA	NA
Additional beverages												
Total FM ^7^	94.53	5.91	0.30	0.92	**<0.001**	−1.49	−0.25	−0.05	0.49	0.122	**<0.001**	0.960
AS ^6^	−3.4	−0.49	−0.07	0.50	0.074	−6.80	−1.13	−0.12	0.89	**0.004**	NA	NA
NAS ^6^	125.08	17.87	0.08	0.90	**0.001**	65.17	10.86	0.07	0.88	**0.006**	NA	NA
Total juice ^7^	−35.72	−2.23	−0.48	0.79	**<0.001**	−5.91	−1	−0.15	0.13	0.484	**<0.001**	**<0.001**
AS ^7^	−16.93	−1.06	−0.08	0.26	**0.042**	−11.71	−1.95	−0.18	0.37	0.201	0.293	**0.002**
NAS ^7^	−43.45	−2.72	−0.40	0.62	**<0.001**	−2.03	−0.34	−0.03	0.03	0.730	**0.001**	**<0.001**
Total KOM ^8^	183,630.43	18,363.04	0.10	0.67	**0.004**	−10.52	−1.75	−0.03	0.50	0.115	**0.012**	NA
SS ^8^	1598.54	159.85	−0.001	0.03	0.626	649.54	108.26	0.001	0.03	0.724	0.669	NA
NSS ^6^	95.38	13.63	0.03	0.11	0.465	−11.25	−1.87	−0.03	0.55	0.092	NA	NA

^1^ Trends were assessed by linear regression in R. For each analysis, the slope of the trend is represented by L/p/y, and significance was defined as *p* < 0.05 (bold). R^2^ is the coefficient of determination. ^2^ Comparisons of the rate of change between two time periods were assessed by fitting segmented linear regression models in R. A significant difference between the two periods was defined as *p* < 0.05 (bold). ^3^ Data available from 2003; trends calculated from 2003 to 2024 where applicable. ^4^ Data available from 2000; trends calculated from 2000 to 2024 where applicable. ^5^ Data available from 2007; trends calculated from 2007 to 2024 where applicable. ^6^ Data available from 2018; trends calculated from 2018 to 2024 where applicable. ^7^ Data available from 2009; trends calculated from 2009 to 2024 where applicable. ^8^ Data available from 2015; trends calculated from 2015 to 2024 where applicable. %/p/per, percentage per person per period; %/p/y, percentage per person per year; L/p/y, litres per person per year; AS, added sugar; CSD, carbonated soft drinks; ED, energy drinks; FM, flavoured milks; FW, flavoured waters; FNC, functional beverages; KOM, kombucha; MX, mixers; NA, not applicable; NAS, no added sugar; NoS, no sugar; NSS, non-sugar sweetened; PW, plain water; SD, sports drinks; SS, sugar-sweetened; TEA, ready to drink teas; UNS, unsweetened; WBB, water-based beverages.

**Table 3:** Sugar content and change in per capita sugar contribution over time for unadjusted sugar content values ^1^ compared to those adjusted based on current nutritional information panel data ^2^.

Beverage Category	Sugars Content (g/100 mL) ^3^			Change in Sugars Contribution (g/p/y) ^4^		Change in Sugars Contribution (%/Period) ^5^	
	1997/2009	2024 Unadjusted	2024 Adjusted	Unadjusted	Adjusted	Unadjusted	Adjusted
Water-based beverages (1997 to 2024)							
Total WBB-SS ^3^	10.8	9.9	8.8	−120	−140	−37.0	−44.0
CSD-SS	11.1	11.1	9.5	−150	−170	−50.5	−57.6
ED-SS	10.9	10.9	11.5	20	30	4278.6	4519.6
TEA-SS	6.7	6.7	4.6	2	2	666.7	426.4
MX-SS	9.7	9.7	7.1	−3	−10	−24.4	−44.7
SD-SS	5.9	5.9	5.9	10	10	258.4	258.4
FW-SS	4.6	4.6	6.5	−1	−0.4	−37.1	−11.1
FNC-SS ^6^	NA	NA	4.0	NA	7	NA	79.3
Additional beverages (2009 to 2024)							
Total ADD-SS ^3^	10.5	10.1	8.8	−40	−50	−20.0	−30.0
Total FM	8.9	8.9	AS: 8.7; NAS: 4.1	21	17	94.7	77.8
Total JUICE	10.7	10.7	AS: 9.7; NAS: 8.9	−60	−70	−35.8	−44.8
KOM-SS ^7^	2.5	2.5	3.0	0.02	0.02	1500.0	1820.0

^1^ Unadjusted sugars content values used in previous analyses based on Brix refractometry of representative beverages. ^2^ Sugars content data were collected for all listed beverages within the 2024 sales dataset where data were available, and the average of these values was used as an adjusted for the sugars content of each beverage category. As the nutrition data were collected in October 2025, these values were used as a proxy to estimate the 2024 sugar content. ^3^ Sugars content estimates for total water-based beverages and total additional beverages were calculated according to the sales-based contributions of each sugars-containing beverage with that grouping. ^4^ Change in sugars contribution in g per person per year (g/p/y) was calculated according to the formula: Change in sugars contribution per capita per year = ((Se − Sb)/n) × 1000, where Se and Sb represent per capita sugar contribution (kg/person) at the end and beginning of each period, respectively, and n represents the number of years in a period. ^5^ Percent change in sugars contribution over each applicable period was calculated according to the formula: Change in sugars contribution over period (%) = ((Se − Sb)/Sb) × 100, where Se and Sb represent per capita sugar contribution (kg/person) at the end and beginning of each period, respectively. ^6^ No unadjusted data was available for functional beverages. The data reflect the time period from 2018 to 2024. ^7^ Data and calculations for kombucha reflect the time period from 2015 to 2024. ADD-SS, sugars-containing additional beverages (including those with intrinsic sugars); AS, added sugars; CSD, carbonated soft drinks; ED, energy drinks; FM, flavoured milks; FW, flavoured waters; FNC, functional beverages; KOM, kombucha; MX, mixers; NAS, no added sugars; SD, sports drinks; SS, sugar-sweetened; TEA, ready to drink teas; WBB-SS, sugar-sweetened water-based beverages.

## Data Availability

Access to the data presented in this study is contingent upon a formal request to the authors due to restrictions on data sharing imposed by the data providers. Data were obtained under license from the Australian Beverages Council and Circana Connect, and access is therefore available from the authors upon reasonable request and with permission of the data providers.

## Supplementary Materials

The following supporting information can be downloaded at: https://www.mdpi.com/article/10.3390/nu18020361/s1. Table S1: Beverage categories and subcategories included for the major datasets, with examples.; Table S2: Beverage categories and sub-categories included within each beverage umbrella grouping; Table S3: Trends in volume sales per capita for each beverage grouping, category, and sub-category for segmented time periods including 1997 to 2014, 2015 to 2024, and 1997 to 2018; Figure S1: Heatmap summarizing the direction and magnitude of trends in volume sales per capita per year (L/person/year, calculated via linear regression analysis) for each beverage grouping, category, and sub-category for the full 28-year time period (1997–2024), as well as segmented time periods: 1997 to 2014, 2015 to 2024, 1997 to 2018, and 2019 to 2024.

## Abbreviations

The following abbreviations are used in this manuscript:

SSB	Sugar-sweetened beverages
NSSB	Non-sugar-sweetened beverages
WBB	Water-based beverages
T2D	Type 2 diabetes
CVD	Cardiovascular disease
NNPAS	National Nutrition and Physical Activity Survey
ABCL	Australian Beverages Council
NSS	Non-sugar sweetened
SS	Sugar-sweetened
UNS	Unsweetened
FNC	Functional beverages
CSD	Carbonated soft drinks
ED	Energy drinks
SD	Sports drinks
TEA	Ready-to-drink teas
MX	Mixers
FW	Flavoured waters
PW	Plain waters
FM	Flavoured milks
KOM	Kombucha
AS	Added sguars
NAS	No added sugars
ADD	Additional beverages
