# Supplementary material for: Evolving Sweet Preferences: Temporal Trends in Australian Non-Alcoholic Beverage Sales from 1997 to 2024
Source: Nutrients. 2026 Jan 22;18(2):361. doi: 10.3390/nu18020361 (PMC12844610; doi:10.3390/nu18020361)
Supplement: Supplementary file 1 [file nutrients-18-00361-s001.zip › nutrients-4081162-supplementary.pdf]

Supplementary Materials

Supplementary Table S1. Beverage categories and subcategories included for the major datasets, with examples.

Beverage category	Beverage sub-category	Previous dataset	Current dataset	Included data (years)	Examples
Primary water-based beverages					
Carbonated soft drinks	Total	✓	✓	1997-2024	All carbonated soft drinks not otherwise classified as mixers, energy drinks, sports drinks, flavoured sparkling waters, ready to drink teas, or functional beverages.
	SS	✓	✓	1997-2024	Regular/full sugar varieties
	NSS	✓	✓	1997-2024	Diet/no sugar sweetened varieties.
Energy drinks	Total	✓	✓	1997-2024	Drinks containing stimulants, primarily caffeine.
	SS	✓	✓	1997-2024	Regular/full sugar varieties
	NSS	✓	✓	2003-2024	Diet/no sugar but sweetened varieties.
Ready to drink teas	Total	✓	✓	1997-2024	Drinks based on iced/cold tea.
	SS	✓	✓	1997-2024	Regular/full sugar varieties
	NSS	✓	✓	2000-2024	Diet/no sugar sweetened varieties.
Mixers	Total	✓	✓	1997-2024	Drinks traditionally intended for mixing with alcoholic beverages, such as ginger ale, tonic water, creaming soda, and lemon, lime, and bitters.
	SS	✓	✓	1997-2024	Regular/full sugar varieties
	NSS	✓	✓	1997-2024	Diet/no sugar sweetened varieties.
Sports drinks	Total	✓	✓	1997-2024	Drinks marketed for sports use, to provide additional carbohydrate and/or electrolytes to support hydration and/or performance.
	SS	✓	✓	1997-2024	Regular/full sugar varieties
	NSS	✓	✓	1997-2024	Diet/no sugar sweetened varieties.
Flavoured waters[1]	Total	✓	✓	1997-2024	Waters with flavours and sweeteners added. Includes both still and carbonated varieties, as well as traditional flavoured mineral waters.
	SS	✓	✓	1997-2024	Regular/full sugar varieties

Beverage category	Beverage sub-category	Previous dataset	Current dataset	Included data (years)	Examples
	NSS	✓	✓	1997-2024	Diet/no sugar sweetened varieties.
Functional beverages	Total		✓	2018-2024	Water based beverages proposing to provide a perceived or actual health benefit beyond basic hydration. Examples include alkaline water, coconut water, nootropic drinks, protein water, cactus water, probiotic sodas, vitamin waters, and wellness waters.
	SS		✓	2018-2024	Regular/full sugar varieties, including coconut water.
	NSS		✓	2018-2024	Diet/no sugar sweetened varieties.
	UNS		✓	2018-2024	Unsweetened varieties
Plain water	Total	✓	✓	1997-2024	Water with no additional health benefit or added ingredients
	Still	✓	✓	1997-2024	Still plain water
	Sparkling	✓	✓	2007-2024	Carbonated plain water
Additional beverages					
Flavoured milks	Total	✓	✓	2009-2024	Beverages based on milk (mammalian or plant-based), including protein drinks, ready to drink iced coffees, and flavoured milks.
	AS		✓	2018-2024	Varieties containing added sugar (in addition to inherent sugar)
	NAS		✓	2018-2024	Varieties not containing added sugar; may contain non-sugar sweeteners
Juices	Total	✓	✓	2009-2024	Fruit juice-based beverages
	AS	✓	✓	2009-2024	Varieties containing added sugar (in addition to inherent sugar). Fruit juice component usually 20-50%.
	NAS	✓	✓	2009-2024	Varieties not containing added sugar, usually labelled as 100% juice. Do contain inherent sugar.
Kombucha	Total	✓	✓	2015-2024	Beverages specifically marketed as kombucha
	SS	✓	✓	2015-2024	Regular/full sugar varieties
	NSS		✓	2018-2024	Diet/no sugar sweetened varieties

[1] Flavoured waters were referred to as mineral waters in the 1997 to 2018 dataset. AS, added sugar; NAS, no added sugar; SNSS, non-sugar-sweetened; SS, sugar-sweetened; UNS, unsweetened.

Supplementary Table S2. Beverage categories and sub-categories included within each beverage umbrella grouping.

**Beverage grouping**	**Abbreviation**	**Description**	**Included categories and/or sub-categories**
Total water-based beverages	WBB	Sum of all WBB categories	CSD, ED, SD, TEA, MX, FW, PW, FNC
Total water-based sugar sweetened beverages	WBB-SS	Sum of all WBB sub-categories sweetened with sugar	CSD-SS, ED-SS, SD-SS, TEA-SS, MX-SS, FW-SS, FNC-SS
Total water-based non-sugar sweetened beverages	WBB-NSS	Sum of all WBB sib-categories sweetened with a non-sugar alternative	CSD-NSS, ED-NSS, SD-NSS, TEA-NSS, MX-NSS, FW-NSS, FNC-NSS
Total water-based unsweetened beverages	WBB-UNS	Sum of all WBB sub-categories not containing a sweetener	FNC-UNS, PW-Still, PW-Sparkling
Total no sugar beverages	WBB-NoS	Sum of all WBB sub-categories not containing sugar, including WBB-NSS and WBB-UN	CSD-NSS, ED-NSS, SD-NSS, TEA-NSS, MX-NSS, FW-NSS, FNC-NSS, PW-Still, PW-Sparkling, FNC-UNS
Total additional beverages	ADD-B	Sum of all additional beverage categories	FM, JUICE, KOM
Total sweet additional beverages	ADD-SS	Sum of all additional beverages sub-categories containing sugar, including intrinsic sugars	FM-AS, FM-NAS, JUICE-AS, JUICE-NAS, KOM-SS

ADD-B, additional beverages; AS, added sugar; CSD, carbonated soft drinks; ED, energy drinks; FM, flavoured milks; FW, flavoured waters; FNC, functional beverages; KOM, kombucha; MX, mixers; NA, not applicable; NAS, no added sugar; NoS, no sugar; NSS, non-sugar sweetened; PW, plain water; SD, sports drinks; SS, sugar-sweetened; TEA, ready to drink teas; UNS, unsweetened; WBB, water-based beverages.

Supplementary Table S3. Trends in volume sales per capita[1] for each beverage grouping, category, and sub-category for segmented time periods including 1997 to 2014, 2015 to 2024, and 1997 to 2018.

**Beverage grouping/ category/ sub-category**	**1997 to 2014**					**2015 to 2024**					**1997 to 2018**				
	**Growth rate**			**Trend**		**Growth rate**			**Trend**		**Growth rate**			**Trend**	
	%/p/per	%/p/y	L/p/y	R^2	p	%/p/per	%/p/y	L/p/y	R^2	p	%/p/per	%/p/y	L/p/y	R^2	p
Water-based beverages															
Total WBB	14.61	0.81	0.87	0.78	**<0.001**	15.89	1.59	2.09	0.92	**<0.001**	23.58	1.07	1.35	0.81	**<0.001**
Total SS	-17.91	-0.99	-0.93	0.97	**<0.001**	-15.88	-1.59	-1.25	0.92	**<0.001**	-26.08	-1.19	-1.02	0.98	**<0.001**
Total NSS	11.09	0.62	0.32	0.60	**<0.001**	41.52	4.15	1.75	0.94	**<0.001**	15.07	0.68	0.24	0.53	**<0.001**
Total UNS	502.41	27.91	1.48	0.91	**<0.001**	47.84	4.78	1.54	0.74	**0.001**	783.69	35.62	2.13	0.87	**<0.001**
Total NoS	89.94	5	1.80	0.93	**<0.001**	45	4.5	3.29	0.96	**<0.001**	138.63	6.3	2.36	0.91	**<0.001**
Total CSD	-19.35	-1.08	-1.10	0.93	**<0.001**	-7.47	-0.75	-0.51	0.55	**0.014**	-27.79	-1.26	-1.35	0.93	**<0.001**
SS	-30.05	-1.67	-1.49	0.96	**<0.001**	-27	-2.7	-1.6	0.96	**<0.001**	-41.19	-1.87	-1.56	0.98	**<0.001**
NSS	16.28	0.9	0.39	0.62	**<0.001**	31.98	3.2	1.09	0.92	**<0.001**	16.85	0.77	0.21	0.32	**0.006**
Total ED	3799.17	211.07	0.37	0.81	**<0.001**	58.72	5.87	0.37	0.94	**<0.001**	4273.78	194.26	0.37	0.88	**<0.001**
SS	3354.61	186.37	0.33	0.8	**<0.001**	19.1	1.91	0.11	0.96	**<0.001**	3632.32	165.11	0.32	0.87	**<0.001**
NSS[2]	6158.73	513.23	0.04	0.81	**<0.001**	376.79	37.68	0.25	0.89	**<0.001**	8930.79	558.17	0.05	0.88	**<0.001**
Total TEA	1145.07	63.62	0.12	0.89	**<0.001**	-28.1	-2.81	-0.08	0.86	**<0.001**	1300.48	59.11	0.12	0.93	**<0.001**
SS	966.19	53.68	0.1	0.89	**<0.001**	-38.05	-3.8	-0.08	0.89	**<0.001**	1115.59	50.71	0.11	0.93	**<0.001**
NSS[3]	702609.33	46840.62	0.02	0.85	**<0.001**	46.37	4.64	0.01	0.31	0.095	726197.3	38220.91	0.02	0.91	**<0.001**
Total MX	-2.57	-0.14	-0.002	0.002	0.837	-5.62	-0.56	-0.02	0.02	0.687	0.48	0.02	0.01	0.09	0.174
SS	-20.79	-1.15	-0.02	0.01	0.762	-1.79	-0.18	0.01	0.01	0.798	-10.88	-0.49	-0.04	0.05	0.324
NSS	11.08	0.62	0.02	0.01	0.78	-7.58	-0.76	-0.03	0.14	0.296	9.00	0.41	0.06	0.09	0.171
Total SD	146.29	8.13	0.15	0.89	**<0.001**	65.84	6.58	0.32	0.95	**<0.001**	196.47	8.93	0.16	0.94	**<0.001**
SS	133.86	7.44	0.14	0.88	**<0.001**	41.97	4.20	0.19	0.94	**<0.001**	178.76	8.13	0.15	0.93	**<0.001**
NSS	6576.34	365.35	0.01	0.53	**<0.001**	521.58	52.16	0.12	0.91	**<0.001**	9365.37	425.7	0.01	0.71	**<0.001**
Total FW	-31.4	-1.74	-0.15	0.7	**<0.001**	30.22	3.02	0.14	0.81	**<0.001**	-29.32	-1.33	-0.10	0.58	**<0.001**
SS	17.59	0.98	-0.002	0.004	0.804	-51.34	-5.13	-0.21	0.73	**0.002**	-17.23	-0.78	0.001	0.001	0.890
NSS	-69.33	-3.85	-0.15	0.8	**<0.001**	324.61	32.46	0.35	0.89	**<0.001**	-38.69	-1.76	-0.11	0.65	**<0.001**
Total PW	502.86	27.94	1.48	0.91	**<0.001**	46.81	4.68	1.49	0.73	**0.002**	782.8	35.58	2.13	0.87	**<0.001**
Still	428.82	23.82	1.2	0.91	**<0.001**	48.56	4.86	1.39	0.74	**0.001**	682.28	31.01	1.81	0.86	**<0.001**
Sparkling[4]	109.43	13.68	0.34	0.94	**<0.001**	33.62	3.36	0.1	0.5	**0.022**	184.33	15.36	0.39	0.97	**<0.001**

**Beverage grouping/ category/ sub-category**	**1997 to 2014**					**2015 to 2024**					**1997 to 2018**				
	**Growth rate**			**Trend**		**Growth rate**			**Trend**		**Growth rate**			**Trend**	
	%/p/per	%/p/y	L/p/y	R^2	p	%/p/per	%/p/y	L/p/y	R^2	p	%/p/per	%/p/y	L/p/y	R^2	p
Total FNC[5]	NA	NA	NA	NA	NA	86.35	12.34	0.25	0.82	**0.005**	NA	NA	NA	NA	NA
SS	NA	NA	NA	NA	NA	80.07	11.44	0.21	0.77	**0.010**	NA	NA	NA	NA	NA
NSS	NA	NA	NA	NA	NA	96.47	13.78	0.005	0.54	0.058	NA	NA	NA	NA	NA
UNS	NA	NA	NA	NA	NA	144.93	20.7	0.04	0.89	**0.001**	NA	NA	NA	NA	NA
Additional beverages															
Total FM[6]	34.74	5.79	0.26	0.96	**<0.001**	36.67	3.67	0.25	0.71	**0.002**	71.87	7.19	0.30	0.98	**<0.001**
AS[5]	NA	NA	NA	NA	NA	-3.4	-0.49	-0.07	0.50	0.074	NA	NA	NA	NA	NA
NAS[5]	NA	NA	NA	NA	NA	125.08	17.87	0.08	0.90	**0.001**	NA	NA	NA	NA	NA
Total JUICE [6]	-21.45	-3.58	-1.06	0.99	**<0.001**	-15.12	-1.51	-0.15	0.30	0.104	-32.84	-3.28	-0.87	0.98	**<0.001**
AS[6]	15.97	2.66	0.31	0.74	**0.029**	-27.21	-2.72	-0.16	0.55	**0.014**	-8.06	-0.81	0.002	0.0001	0.978
NAS[6]	-36.84	-6.14	-1.37	0.98	**<0.001**	-5.66	-0.57	-0.01	0.01	0.771	-43.03	-4.30	-0.87	0.89	**<0.001**
Total KOM[7]	NA	NA	NA	NA	NA	183630.43	18363.04	0.10	0.67	**0.004**	93078.29	23269.57	0.11	0.74	0.141
SS[7]	NA	NA	NA	NA	NA	1598.54	159.85	-0.001	0.03	0.626	-80.92	-20.23	0.01	0.05	0.767
NSS[5]	NA	NA	NA	NA	NA	95.38	13.62	0.03	0.11	0.465	NA	NA	NA	NA	NA

[1] Trends were assessed by linear regression in R. For each analysis, the slope of the trend is represented by L/p/y and significance was defined as $p < 0.05$ (bold). R^2 is the coefficient of determination. [2] Data available from 2003; trends calculated from 2003-2024 where applicable. [3] Data available from 2000; trends calculated from 2000-2024 where applicable. [4] Data available from 2007; trends calculated from 2007-2024 where applicable. [5] Data available from 2018; trends calculated from 2018-2024 where applicable. [6] Data available from 2009; trends calculated from 2009-2024 where applicable. [7] Data available from 2015; trends calculated from 2015-2024 where applicable. %/p/per, percentage per person per period; %/p/y, percentage per person per year; L/p/y, litres per person per year; AS, added sugar; CSD, carbonated soft drinks; ED, energy drinks; FM, flavoured milks; FW, flavoured waters; FNC, functional beverages; KOM, kombucha; MX, mixers; NA, not applicable; NAS, no added sugar; NoS, no sugar; NSS, non-sugar sweetened; SD, sports drinks; SS, sugar-sweetened; TEA, ready to drink teas; UNS, unsweetened; WBB, water-based beverages.

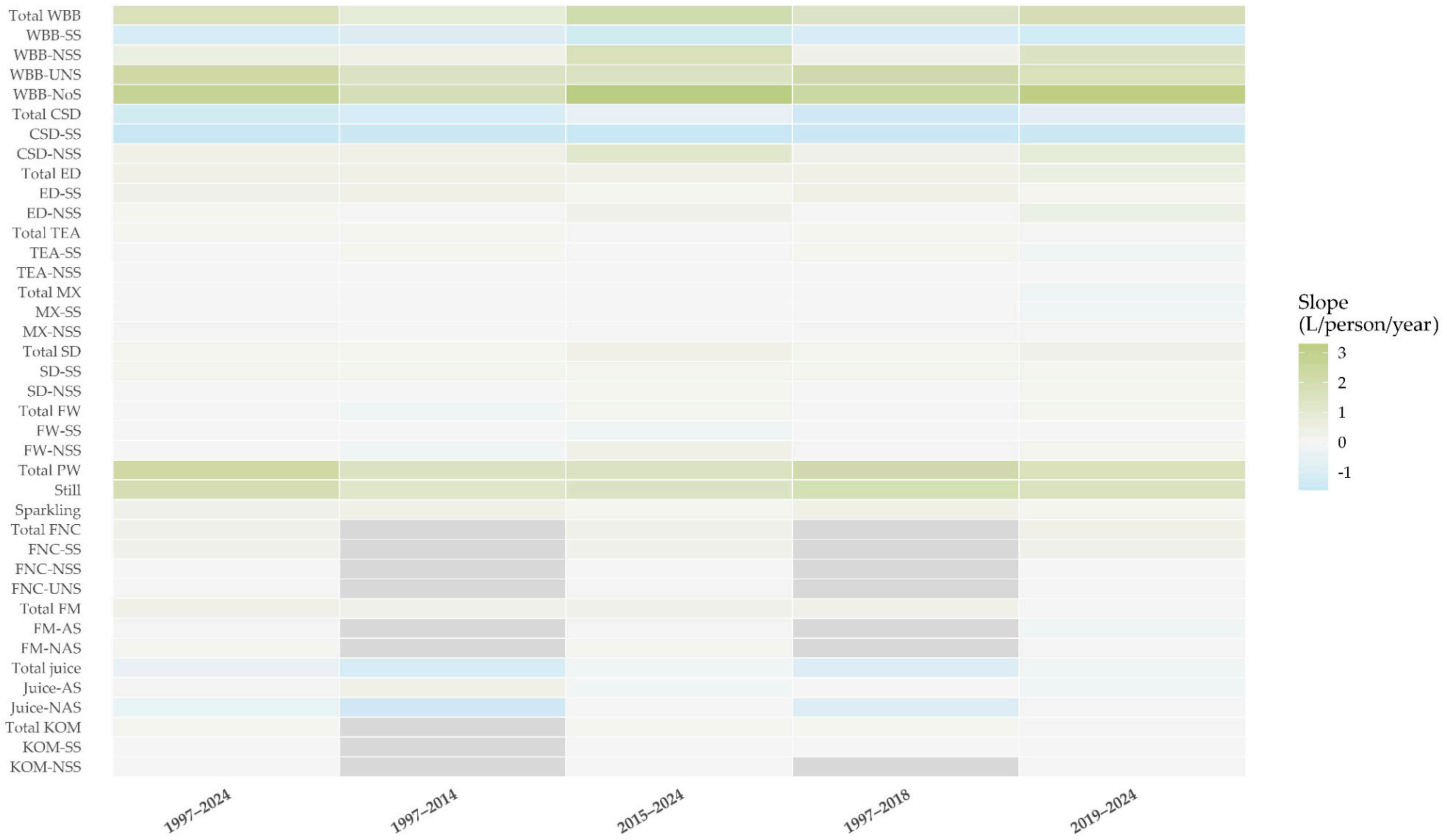

Supplementary Figure S1. Heatmap summarizing the direction and magnitude of trends in volume sales per capita per year (L/person/year, calculated via linear regression analysis) for each beverage grouping, category, and sub-category for the full 28-year time period (1997-2024), as well as segmented time periods: 1997 to 2014, 2015 to 2024, 1997 to 2018, and 2019 to 2024. Linear regression analysis and heatmap construction were performed using R. Grey boxes indicate where no data were available to assess the trend for that time period. Data for the full time period (1997 to 2024) reflects the available data for each beverage grouping, as detailed in **Supplementary Table S1**. AS, added sugar; CSD, carbonated soft drinks; ED, energy drinks; FM, flavoured milks; FW, flavoured waters; FNC, functional beverages; KOM, kombucha; MX, mixers; NA, not applicable; NAS, no added sugar; NoS, no sugar; NSS, non-sugar sweetened; SD, sports drinks; SS, sugar-sweetened; TEA, ready to drink teas; UNS, unsweetened; WBB, water-based beverages.
